# Search Dog Handlers Show Positive Bias When Scoring Their Own Dog's Performance

**DOI:** 10.3389/fvets.2020.00612

**Published:** 2020-09-10

**Authors:** Corinna C. A. Clark, Nicola J. Sibbald, Nicola J. Rooney

**Affiliations:** ^1^Department of Life Sciences, University of Warwick, Coventry, United Kingdom; ^2^Defence Science and Technology Laboratory, Salisbury, United Kingdom; ^3^Animal Welfare and Behaviour Group, Bristol Veterinary School, University of Bristol, Bristol, United Kingdom

**Keywords:** bias, rating, working dog, leniency, reliability, validity

## Abstract

Self-assessments of performance are commonly used in the human workplace, although compared to peer or supervisor ratings, they may be subject to positive biases or leniency. The use of subjective ratings scales in animal sciences is also common, although little consideration is usually given to possible rater bias. Dog handlers, work very closely and form strong relationships with their dogs and are also best placed to monitor dog performance since they often work in isolation. Previous work found ratings of search dog performance correlated well between experienced dog trainers, instructors, and scientists; but until now, there has been no investigation into ratings made by a dog's own handler. We compared handlers' subjective assessment of their own dog's search performance to scores given by other handlers and in a second study, to scores made by impartial raters. We found that handlers generally showed leniency; for example scoring their own dogs more favorably for Control (responsiveness to commands) and Strength of Indication. But the degree of bias varied with the trait being scored and between raters. Such differences may be attributable to greater desirability or importance of favorable scores for certain traits, or a lack of clarity of their precise meaning. Handlers may vary in susceptibility to bias due to differing levels of experience and the extent to which they view their dog's ability as dependent on their own. The exact causes require further investigation. We suggest working dog agencies provide rater-training to overcome leniency, improve reliability and validity, and to increase handler's motivation to provide accurate assessments. This study represents one of a series of steps to formulate robust, validated and evidence-based performance rating systems and has relevance to any situation where raters assess their own performance or others (particularly where they may have a vested interest in, or loyalty toward, the ratee).

## Introduction

Search dog teams perform a vital role in law enforcement agencies, search and rescue teams and in the military, searching for targets as diverse as people, drugs, money, weapons, and explosives. Many consider them to be, if not the most effective method [e.g., (1)], the fastest and most versatile method of detecting explosives (2). Specialist medical detection dogs are also effective in aiding the control of chronic life-threatening conditions by alerting their owners to physiological changes, such as hypoglycaemia in patients with diabetes (3, 4). To maintain standards of performance and maximize search/detection capability, organizations often monitor, and record various aspects of daily operational performance. This monitoring process is essential to address short-term training needs and for longer-term planning and changes in policy. For example longitudinal data can help to formulate optimal rearing and training protocols, to assess the impact of changes in care or operational procedures (e.g., work rest patterns, climate acclimatization), as well as answering questions such as whether certain breeds are better suited to a particular type of work or environment. If critical decisions are to be based on such data, it is imperative that the collection is robust, validated and evidence-based. Hence we have embarked in a multi-stage process to develop an optimal data collection tool for military search dogs, which rates the most critical behavioral traits and presents them in the optimal way (5). One stage of development is described in this paper.

Due to the nature of the tasks being performed (e.g., military dogs working in combat zones and search and rescue dogs working in remote or unstable terrain) handlers frequently work singly and so the responsibility for measuring performance will largely fall to the handlers themselves. For performance monitoring to be effective in these circumstances, methods need to be practically feasible and provide timely, accurate, and reliable information. Suitable behavioral measures of search performance have been derived by Rooney et al. (6) and it has been demonstrated that subjective scales rating these characteristics can be an effective method of point sampling search performance in an experimental setting and monitoring performance longitudinally using trainers' ratings (7). Subjective ratings of this kind have the advantage of being relatively quick and easy, so not only can they be completed in the field but feedback on changes in performance can be instantaneous.

Subjective assessments can, however, be subject to rater error or bias [e.g., (8–11)]. There are many forms and sources of bias, but particularly relevant in this setting are biases where raters provide more positive scores than reflect real performance, because they are either rating themselves or a colleague/friend. Positive response biases to survey questions and subjective rating scales are commonly reported, although they can have differing motivations, being described using terms such as leniency (12, 13), acquiescence (14, 15) and satisficing (11, 16). In surveys, more than 50% of respondents frequently believe themselves to be “above average” in respect to whatever question is being asked (17–19). Self-report bias, or leniency, when raters assess and score their own performance in a task is well-documented [e.g., (19–21)]. Although dog handlers are not scoring their own performance, they are working closely with their dog as a single search team and so it is conceivable that they may be reluctant to give poor scores to their own dog, for fear that it will reflect badly on their own performance, or out of “loyalty” to the dog akin to the friendship bias or “own-group” bias seen in peer assessments (22, 23). Handlers may also be influenced by an a-priori expectation of the dog's capabilities based on previous experience (e.g., my dog would usually perform better than this, so I will give him the benefit of the doubt on this occasion), leading to more lenient marking. This is important if procedural decisions are to be made on the basis of handlers' performance ratings.

We examined potential leniency in a group of operational dog handlers, testing whether subjective assessment of their own search performance was more favorable by comparing the scores they gave to their own dog's search performance to scores given by other handlers (Study 1) and independent raters (Study 2). Initially, 12 arms and explosives search (AES) dog handlers were divided into pairs and asked to rate the performance of their own and their partner's dog. As it would not be practically feasible to carry out experiments in an operational environment and having previously demonstrated that observers can reliably rate dogs' ability from video recordings (7), we used video recordings of training searches. Handlers scored several performance measures relevant to AES dogs (7), as well as giving a score for Overall Ability. This allowed us to test whether handlers showed rank order consistency (i.e., best to worst performance) over a series of searches and whether scores given to their own searches were more favorable than those given by the other handler in the pair. As any differences in overall ratings between own and other scores could be attributable to bias in either party, in Study 2 we compared own and other handler scores to independent expert ratings to clarify which were more accurate (i.e., are closer to the true score), the expert ratings were assumed to be an unbiased reflection of actual performance. We hypothesized that handlers would rate their own searches differently, and that in general, score their own dog more favorably (or leniently) compared to other handers and experts' scores.

## Methods

### Study 1

#### Subjects and Training Searches

Twelve trainee arms and explosives search (AES) dog handlers were recruited. Each had between 1 and 10 years (average 4 years) experience of handling dogs and all were in their final week of a 15-weeks training course with their AES dog. Each handler had been filmed (using a hand-held video camera with a wide-angle lens) performing training searches on ten occasions during the previous 7 weeks. A section of each search was selected which was clear to see and which together showed a wide variety of different levels of performance. Cropped sections varied from 6 to 16 min long (average = 12 min). The majority of searches included the dogs encountering an explosives training sample (104 searches with, 16 without), and the recording ended after the dog alerted to or missed the hide.

#### Video Observations

Each of the 12 handlers was randomly paired with another handler at the same stage of their training, but who had been in a different training group and hence they had rarely seen each other search. Handlers watched and rated the videos in these pairs, with observations split into two sessions on consecutive days to reduce fatigue. In each session, handlers watched five of their own and five of their partner's searches, so they scored 10 own and 10 other searches in total. Thus, each of 120 video clips were watched by two handlers; generating one “own” handler score and one “other” handler score per dog, per characteristic.

The order of searches was randomized, but alternated between the two handlers. Immediately prior to observations, pairs were advised that they should: observe the entire video before rating performance; base their ratings only upon what they had seen on the video; and try to use the full range of the rating scales if appropriate. An experimenter was present throughout to ensure that handlers did not talk to each other about their ratings. There was a pause after each film to allow handlers as much time as they needed to complete the ratings form and short breaks after the third, sixth and eighth films.

#### Performance Measures

Previous work had prioritized the most relevant dimensions for current AES performance (7), from which the following seven characteristics were chosen:

**Control**, or response to commands;

**Motivation** to search;

**Stamina** throughout the search;

**Confidence** in the environment;

**Independence** or ability to search without direction;

**Distraction** from searching;

Strength of (behavioral) **Indication** when the dog locates a hide.

Handlers rated the characteristics on a 1 to 5 scale: 1 = very low level of the characteristic; 2 = low; 3 = intermediate; 4 = high; 5 = very high, recording this on a pre-printed sheet (tick boxes). Beyond a brief instruction on the meaning of these terms, no further descriptors, or guidance for marking was given. The scales were explicitly not valenced (very low to very high, as opposed to very poor to very good) and handlers were instructed that their scores should reflect the amount of the trait present and not how well the dog was performing. However, in general, high scores for a particular trait would indicate a well-performing dog (e.g., Control and Motivation). Exceptions to this were Independence and Distraction: previous work has indicated that some handlers view ideal levels of Independence as being a score of 3 or 4, rather than 5 (6); positive bias in Distraction would be evidenced by low scores, as high scores indicate a very distracted dog, which is not desirable. Handlers were also asked to give a clearly valenced score for Overall Ability out of 10, with one being the worst and 10 being the best performance possible.

### Study 2

#### Subjects and Training Searches

A different cohort of nine trainee explosives search dog handlers to those in Study 1 (but at a similar point in training) were filmed, each performing an identical training exercise with their AES dog in which they searched an area for up to 15 min, aiming to locate an explosives training aid.

#### Video Observations

The same performance measures, briefing, and protocol were followed as in Study 1 (see above), with the exception that all nine searches were watched by all nine handlers (in groups of three). Thus, there were nine “own” search scores and 72 “other” for each characteristic of performance per search-team. Impartial expert ratings were obtained from three independent raters: one dog trainer and team instructor with extensive experience assessing performance, and two experimenters experienced in rating dog performance using the scales. Due to their impartiality and for simplicity, we refer to these as “experts” although some of the handlers also had high levels of experience. Expert raters were blind to the scores given by the handlers.

### Statistical Methods

The data were analyzed using non-parametric methods in IBM SPSS statistics 19. Scores were categorized as “own” (the handler rating their own dog's performance) or “other” (rater was not the handler in the clip) or “expert” (Study 2 only).

#### Study 1

To assess whether handlers agreed in their rankings of search performances from best to worst (irrespective of absolute score) we used Spearman's rank order correlation coefficient (*r*_s_), comparing all ratings for own and other scores (across all 120 searches) and within pairs (20 searches per pair). Values of *r*_s_ > 0.7 were taken to indicate a strong association; 0.6–0.7 a good association; 0.5–0.6 moderate and 0.3–0.5 a weak association.

Wilcoxon signed ranks statistic (z) was used to test whether the magnitude of scores from own and other handlers, for each dog, differed significantly.

#### Study 2

Mean “other” and “expert” scores per behavior per dog were produced. We used mean values as medians frequently masked variation between ratings; mean other handler scores for Confidence, for example, varied between 2 and 5, whereas median scores were 4 for all dogs, thus preventing any correlational analysis. Kendall's Coefficient of Concordance (w) was used to compare own, other and expert categories, and Spearman's correlation coefficients calculated for pairwise comparisons.

Friedman test (*T*_F_) was used to check for overall differences in the magnitude of scores between the three categories of rater, using mean other and expert ratings. Pair-wise Wilcoxon signed rank tests were then used to determine which categories of rater differed significantly. We also tested within the expert-rater category whether raters differed from one another to assess the value of their scores as a “gold standard.”

## Results

### Study 1

When considering ratings for all pairs together, there was moderate agreement in scores for Control and Overall Ability, and weak agreement for Motivation, Distraction and Stamina and little agreement in scores for Confidence, Independence and Indication (Table 1). There was however, considerable variability in the level of agreement within pairs, with some pairs in closer agreement across traits than others: pair 1 for example showed good or strong agreement for three traits, whereas pair 4 only agreed (>0.6) for one trait. The likelihood of agreement not only differed between pairs, but also depending on the behavior being scored: for Distraction, for example, pairs 1, 3, and 5 showed good agreement, whereas the other three pairs showed little to no agreement. Rater pairs were more likely to show good agreement when scoring Control and Overall Ability (despite the latter being scored out of 10), with the poorest levels of agreement for Independence.

**Table 1:** Study 1: Agreement between own handler and other handler scores (all ratings, *N* = 120).

	**Control**	**Motivation**	**Stamina**	**Distraction**	**Confidence**	**Independence**	**Indication**	**Overall ability**
Own/other (all ratings)	0.539	0.486	0.372	0.417	0.025	0.263^e^	0.282^f^	0.529^g^
Pair 1	**0.783**	0.480	0.257	**0.607**	0.312	0.525^a^	−0.379^a^	**0.617**
Pair 2	**0.683**	0.354	**0.607**	0.113	−0.334	0.300	0.414^d^	0.584
Pair 3	0.556	0.365	0.479	**0.622**	−0.061	−0.007	0.191^b^	**0.661**^**a**^
Pair 4	0.351	0.239	−0.026	0.279	−0.338	0.055	**0.601**^**a**^	0.272
Pair 5	0.577	0.212	0.067	**0.652**	−0.425	−0.112^a^	0.342^c^	0.518
Pair 6	0.354	**0.756**	0.385	0.099	0.362	0.118	0.494^b^	0.439

*Correlation coefficients between handlers in each pair (Spearman's rho, 2-tailed, N = 20, unless stated otherwise) for each trait. Moderate agreement (>0.5) shaded and good agreement (>0.6) in bold*.

*Where N <20 within pairs or <120 for overall comparison*.

a N = 19;

b N = 17;

c N = 16;

d N = 15;

e N = 118;

f N = 103;

g *N = 119*.

Handler's own scores for Overall Ability were significantly higher than were other handler's scores (Table 2). Handlers also generally scored their own dogs more highly for Control, Motivation, Stamina, Confidence, and Indication; as well as tending to score their own dogs lower (more favorably) for Distraction. As with agreement in rank ordering, whether scores differed significantly varied between pairs and behavioral measures (Table 3). For example, five handlers scored their own dog as having significantly higher levels of Motivation, whilst seven did not. There was general disagreement between own and other handlers in scores for Independence, but no clear pattern of favorable marking as three handlers scored their own dog significantly higher and three significantly lower than the other handlers.

**Table 2:** Study 1: Median scores given by handler for own dog's performance and scores given by other handler and Wilcoxon Signed Ranks statistic (z) comparing within dog, across all 12 handlers.

**Difference**	**Control**	**Motivation**	**Stamina**	**Distraction**	**Confidence**	**Independence**	**Indication**	**Overall ability**
*Z*	−2.658^**^	−3.251^**^	−3.390^**^	1.858 ^p = 0.063^	−2.726^**^	−1.147	−2.853^**^	−3.236^**^
Median score given to own dog	3.5 (4)	3.9 (4)	4.1 (4)	2.0 (2)	4.3 (4)	3.9 (4)	3.9 (4)	7.1 (7)
Median score given to other dog	3.2 (3)	3.6 (4)	4.0 (4)	2.2 (2)	4.1 (4)	3.8 (4)	3.6 (4)	6.8 (7)

** *p < 0.01*.

**Table 3:** Study 1: Significant differences within pairs of handlers for each trait as shown by Wilcoxon Signed Rank tests.

**Difference**		**Control**	**Motivation**	**Stamina**	**Distraction**	**Confidence**	**Independence**	**Indication**	**Overall ability**
Pair 1	1			own*	other*			other*	
	2	own*						own**	own*
Pair 2	3								
	4				other*				
Pair 3	5						other*	other*	
	6		own*			own**	own**	own*	
Pair 4	7		own*	own*					own*
	8	own*	own*		other*		own**	own*	own*
Pair 5	9					other*	other*		
	10		own**	own*					
Pair 6	11	own**	own**				other**		
	12					own*	own*	own*	own*

Own denotes the dog's handler scored them significantly higher, other denotes the other handler rated the dog higher (p < 0.05*; p < 0.01**).

### Study 2

Considering all categories of rater (own, other, expert) there was moderate to strong agreement for most behavioral traits (Table 4) (>0.5), with weak agreement (<0.5) for Motivation, Stamina, and Overall Ability. Pairwise correlations between the categories of rater, indicate that for Distraction, Independence and Indication, agreement was only between other and expert raters; and in general, agreement between own and other, and own and expert, scores was poor (and lower than that between other and expert scores).

**Table 4:** Study 2: Levels of agreement between scores given by own handler, other handlers, and experts.

**Behavior**	**Agreement in rank score**			
	**Own/other/** **expert**	**Own/** **other**	**Own/** **expert**	**Other/** **expert**
Control	**0.774**	0.587	**0.724**	**0.676**
Motivation	0.460	0.331	0.135	0.110
Stamina	0.443	0.191	0.187	0.129
Distraction	**0.678**	0.179	0.448	**0.906**
Confidence	**0.765**	0.470	**0.878**	**0.619**
Independence	**0.628**	0.184	0.370	**0.715**
Indication	0.587	0.393	0.217	0.519
Overall ability	0.403	−0.028	−0.113	0.421

*Kendall's coefficient of concordance (W), for 3 -way comparison and Spearman's rho (r_s_) for pairwise comparisons. Moderate agreement (>0.5) shaded and good agreement (>0.6) in bold*.

**Figure 1:**
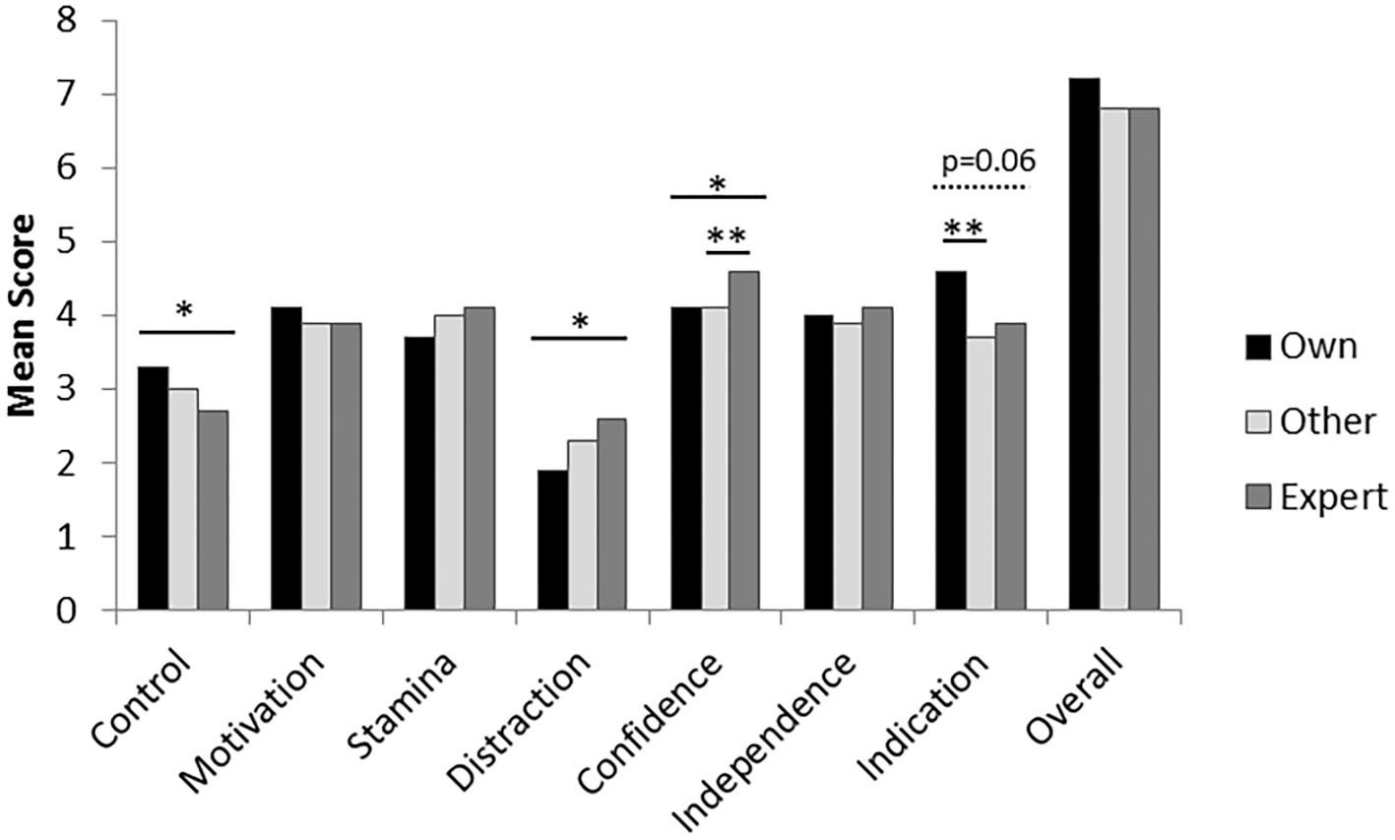
Differences between own (nine raters), mean of other handler (*n* = 8) and expert (*n* = 3) ratings for the performance traits. Asterisks denote significant differences seen between specific raters, using pair-wise Wilcoxon Signed Ranks tests (*p* < 0.05*; *p* < 0.01**).

Handler's own scores were significantly higher than mean expert scores for Control and they tended to be higher for Indication (*p* = 0.06), but were lower for Distraction and Confidence (Figure 1). Other handler scores only differed significantly from experts for Confidence.

There was no significant difference between expert raters in their scores for any of the traits, with the exception of Indication, where one expert rater gave significantly higher scores than both of the other raters (*T*_F_ = 8.12, *p* = 0.017).

## Discussion

In Study 1, handlers generally rated their own dog more favorably than the other handlers, supporting the hypothesis that they exhibited leniency. This was true for all behaviors, except Independence (ability to search without direction), which some handlers rated as higher and others rated lower in their own dogs. This may be because handlers do not see very high levels of Independence as beneficial (6), whereas for all the other behaviors the higher the level of the trait (e.g., Control) the better (except Distraction where the opposite is true). Scores were significantly higher for Control (response to commands), Motivation to search, Stamina throughout the search, Confidence in the environment.

In Study 2 overall agreement in the scoring of behaviors was considerably better than that in Study 1, although pairwise correlations indicated that the improvement was most likely due to agreement between the experts and other handlers. Handlers in the second study also showed favorable marking toward their own dog, particularly for levels of Control, Distraction, and Indication. Interestingly, the expert raters scored Confidence in the environment significantly higher than both own and other raters.

Across both studies the level of agreement differed both between raters and between traits, but in general, where there was a difference between raters it seemed to be the result of more favorable scoring by the handler toward their own dog. Hence, handlers have a tendency to be lenient when assessing (or at least when scoring) their own dog's performance. As the same group of handlers show good agreement with experts when applying the rating scale to other handlers' dogs, yet poor agreement with experts when applying the same scales to their own dogs, this shows that they are not applying the same rating principles when assessing their own and other dogs.

### Leniency Bias Did Not Affect All Behaviors Equally

Although we found considerable evidence for a leniency bias, the effect was not universal across all performance measures (nor all raters) and there could be several reasons for this.

#### Ability to Understand the Trait Being Measured

Some characteristics of performance are likely to be harder to rate accurately than others and we would expect greater agreement where behaviors are inherently easier to interpret, as there should be less variation between handlers and also less uncertainty within-raters in how to apply the 1–5 scale on repeated occasions. For example, Control (response to commands) is a relatively easily quantifiable trait and was the most universally comparable between raters. Independence (ability to search without guidance) on the other hand, showed little agreement. If handlers had a similar understanding of the concept and were marking their own dog's searches more leniently, we might still see agreement in ranking from best to worst, as well as more favorable scoring for their own dog's searches; which seemed to be the case for scores for Control.

Several behavior traits in Study 1 showed poor agreement whilst still being scored more favorably by own handlers (e.g., Motivation, Stamina, Confidence, and Indication). We deliberately chose raters with no experience of using the rating scales; however, they may have struggled to rate searches accurately because they didn't understand the traits, or the variation between the five levels of performance within each trait. A lack of understanding of the trait could lead to careless rating or resorting to particular response styles; for example, a net acquiescence response style (14), where a handler scores their own dog at an above average level (but not the highest level) for every search regardless. A lack of agreement between own and others' ratings can be further exaggerated by ambiguity in the rating scales themselves (24) which can lead to raters scoring ambiguous traits in their own favor (19). This may be reduced by providing raters with more information on each of the traits.

While a lack of understanding of the traits may have been partly responsible for some bias, it seems unlikely to be the only reason. In Study 2, agreement between expert scores and the scores given to other handlers' searches was very good across most traits; however there was very poor agreement between the scores that the same group of handlers gave their own dog's searches and the expert scores. In this study, Distraction (from searching) and (strength of) Indication appeared to be readily quantifiable when handlers rated other dog's searches, as they correlated well with (and did not differ significantly from) expert scores, but were particularly vulnerable to bias when rating their own dogs. This effect has been documented in the field of human psychology, with ratings of own performance frequently overestimated, hence leading to greater agreement in work-place performance assessments between peers and supervisors compared to self-peer and self-supervisor ratings (19, 24). Whilst factors related to the scale design are important and can exacerbate this effect, the psychological processes involved in this optimism or over-estimation of own ability are complex and beyond the scope of this paper [see review (19) and “Bias did not affect raters equally” below]. Our findings do, however, suggest that ratings are biased in a similar way as would be expected if handlers rated themselves, potentially as a result of the closeness of the relationship between dog and handler.

It could be argued that the handlers are not lenient, just more familiar with the dog and hence better able to rate its performance. However, given that one of the experts had also trained all the dogs, and that all significant differences in the scores relative to experts and others were in the direction predicted by leniency, we consider this unlikely. Further studies on the effects of training handlers to provide accurate ratings, would now be valuable.

#### Desirability of Favorable Scores

The relative desirability for a high score within a given trait is also likely to influence how susceptible a measure is to bias (25). For example, we hypothesize that handlers would like their dogs to be maximally obedient and score 5 out of 5 for Control, whereas the ideal score for Independence may in fact be 3 or 4 out of 5. A combination of confusion between raters in what is meant by “Independence,” as well as varying opinions on what constitutes the ideal level of the trait (26), may explain why some handlers (Study 1) rated their own dog significantly higher for this trait, and others significantly lower.

Overall Ability is the one measure where participants do not score how much of a trait is present, but how well they have subjectively assessed that the dog performed. Because Overall Ability scores are subjective and clearly valenced (higher scores are more desirable), we would have predicted this measure to show considerable rater bias. Yet, whilst handlers appeared to score their own dogs more highly in both studies, this was only significant in Study 1. Agreement was low to moderate, which may be a consequence of the greater number of scale options (one to 10 scale, as opposed to the 1–5 scales used for the other traits), or a result of the differing relative importance that handlers assign to the individual component characteristics of performance. It may be that because the scale is so clearly valenced, handlers were reluctant to use the whole scale (including the extremes of the scale) for rating either theirs or other handlers' searches. For example only 5% of Overall Ability scores in Study 1, and 7.4% in Study 2, were below five and while it may be that the searches were all of an above average standard, the existence of a net acquiescence bias (or an avoidance of scale extremes) cannot be discounted. One consequence of net acquiescence across all raters is “range restriction,” whereby only part of a scale is utilized, which can undermine the validity and reliability of results (27). Net acquiescence may also be responsible for the experts' higher scores for Confidence in Study 2: experts were more likely to score dogs at 5 out of 5 (64% of searches), compared to handlers scoring either their own (22%) or other handlers' (22%) searches.

### Bias Did Not Affect Raters Equally

The psychological processes underlying leniency bias are complex and raters may be naïve to their own bias. For example, raters are often able to see bias in the scoring of others, and yet insist that their own ratings are error-free (18). It was clear that not all handlers showed the same degree of bias in scoring. Relative competencies and knowledge are important in producing accurate self-ratings (19); thus, the differences we found may reflect disparity between raters in their understanding of the traits used, or a reliance on rating response styles. These could, in turn, be a result of differing levels of experience and understanding of what constitutes ideal performance (6). It would also interesting to investigate the effect of level of experience on the tendency to be lenient but within this study, although there was variation in handler experience, sample sizes were too small to investigate its effect on rating agreement.

The impact of ratee characteristics on rating ability is well-known in the social science literature [e.g., (28)]. Interest in completing rating tasks, the relevance to the rater, and the perceived importance or consequence of providing accurate ratings are all important motivating factors (12, 17, 29). The raters' personality type (30, 31), their affective state or mood (32), or, in this situation the ratees perception of the “team,” such as the level of attachment between handler and dog and the extent to which they see the dog's performance as reflection of their own ability, may all influence the degree of positive bias. The relative impact of some of these factors on ratings provided by search dog handlers is still to be investigated.

### Consequences for Performance Measurement

To ensure that the data collected is reliable, it is important to ensure that the performance monitoring process is as objective as possible and without bias, whilst also remaining practically feasible. Positive bias will impact on the validity of information collected using subjective scales, which has implications to any situation where data is reliant on subjective ratings, not just the measurement of working dog performance. Leniency may be particularly important when raters have a vested interest in the outcome, but even where this isn't the case, there may be issues with other biases, such as net acquiescence and a reluctance to use the whole scale. Hence, if rating scales are to be used effectively then efforts must be made to check for, and to overcome, biases.

Several measures can be undertaken to reduce the effect of bias. Scales should initially be validated to assess whether some components are more prone to bias. Improving scale design (14), for example providing scale benchmarks (33) may help to improve understanding of the dimensions being measured and the value of benchmarking has been investigated for these scales (5). Using statistical methods to adjust data (35) or partition error variance (36) could be considered, although caution should be exercised when manipulating data [see (37)], especially where bias is not universal across all measures or raters. Care must also be taken to ensure that any supposed bias is not, in fact, an accurate reflection of a skew in the population being measured (i.e., low natural variation in performance). Understanding differences between raters and the occurrence of response styles is also important and rater training may help to simultaneously reduce bias and increase motivation to provide accurate ratings (38).

## Conclusions

Dog handlers showed favorable scoring, or leniency, for several traits of search performance. The degree of bias varied with the trait being scored and also between raters. Raters showed variation in agreement suggesting that they differed in their understanding of the meaning of the traits being measured, although rater bias may have been partly responsible as handlers agreed with expert ratings when assessing other handlers' dogs. Improvements therefore need to be made to ensure the reliability and validity of ratings if they are to be made by lone working handlers. We believe this will be achievable through effective methods of training handlers to rate dogs objectively, potentially both reducing bias and improving understanding and thereby consistent use of scales. This study, whilst using search dog handlers, has relevance in any situation where raters must assess the performance of others, particularly where they may have a vested interest in, or loyalty toward, the ratee.

## Data Availability Statement

The datasets presented in this article are not readily available because they contain information on military working dog performance and are thus sensitive. Requests to access the datasets should be directed to the corresponding author.

## Ethics Statement

Ethical review and approval was not required for the study on human participants in accordance with the local legislation and institutional requirements. Written informed consent for participation was not required for this study in accordance with the national legislation and the institutional requirements. The animal study was reviewed and approved by University of Bristol, Animal Welfare & Ethical Review Board. Written informed consent was obtained from the owners for the participation of their animals in this study.

## Author Contributions

NR and CC conception of idea and preparation of manuscript. CC, NS, and NR methods development. CC and NS data collection and analysis, with input from NR. All authors contributed to the article and approved the submitted version.

## Conflict of Interest

The authors declare that the research was conducted in the absence of any commercial or financial relationships that could be construed as a potential conflict of interest.
